# *Journal of Medical Case Reports*' policy on consent for publication

**DOI:** 10.1186/1752-1947-4-173

**Published:** 2010-06-07

**Authors:** Michael R Kidd, Iain Hrynaszkiewicz

**Affiliations:** 1 Executive Dean, Faculty of Health Sciences, Flinders University, Australia; 2 Honorary Professor, School of Medicine, The University of Sydney, Australia; 3 Managing Editor, BioMed Central, 236 Gray's Inn Road, London WC1X 8HL, UK

## 

*Journal of Medical Case Reports *was launched with the aim of increasing the publication of case reports in the medical literature, because accurate reports of clinical experience are an essential part of the continued development of modern medicine [1]. All the journal's content is highly visible as it is available freely by online open access and indexed in a number of bibliographic databases. With transparency comes responsibility, and we are therefore revising our policy on obtaining consent to publish from the people described in case reports published in the journal to further enhance the protection of privacy.

The practice of obtaining informed consent from people who participate in research has long been established; obtaining informed consent to publish information about individuals is a more recent, but increasingly accepted, concept, which has been embraced by our journal.

An individual's right to privacy is ingrained in the laws of numerous countries, and protection of information obtained as part of the clinician-patient relationship is an essential part of many of the codes of ethics and professional conduct followed by clinicians, researchers, journal editors and publishers around the world [2-4].

The relevant privacy legislation in the United Kingdom, where *Journal of Medical Case Reports *is published, is the Data Protection Act [5]. A key consideration as to whether the Act applies to published articles is whether the data being presented are "personal" or not. If published information is anonymous to the extent that the individual concerned (or anyone who knows them) cannot identify themselves from the published article then the Act does not apply. The Act does also not apply to deceased individuals.

In research involving groups of individuals, the clinical data being analyzed are likely to be presented in summary or aggregated form, and so it may be possible to confidently remove risks to privacy. But this is not possible for medical case reports as these provide detailed descriptions of an individual's medical history.

*Journal of Medical Case Reports *has always striven to protect privacy, and maintains its endorsement of the privacy policies of the International Committee of Medical Journal Editors [2] and the Committee on Publication Ethics [3]. Therefore we have required that authors obtain signed consent for publication for all case reports submitted to the journal, except for rare examples where authors have been able to demonstrate that the person and/or their next of kin are deceased or impossible to trace.

But obtaining consent to publish does not circumvent the need to protect anonymity. The codes of ethics followed by *Journal of Medical Case Reports *go further than data protection legislation in that they require that consent be obtained *and *that only information scientifically relevant to the case being presented be published. So even in the presence of consent, authors and editors are required to ensure that non-essential, and possibly identifying, information about individuals is not included in case reports. Moreover, we have always required that privacy of deceased people is protected and that, wherever possible, consent of the person's next of kin be sought. We recognize that it is not always possible to obtain consent. Where people and their families are non-traceable, deceased, or both, we have provided criteria for publication without consent, which includes ensuring case reports are anonymous.

However, it is clear that articles including three or more indirect identifiers relating to an individual could, theoretically, present risks to privacy [6,7]. To be of value to our readers, a case report published in *Journal of Medical Case Reports *must include at a minimum the age, gender and ethnicity of the individual described in the case report (plus many other clinical details in the majority of reports).

Therefore, our policy on obtaining consent to publish is now mandatory for all cases that relate to a living individual. For case reports that relate to a deceased person, authors must ensure they have met all three criteria specified by the Committee on Publication Ethics' Code of Conduct. Our instructions for authors have been updated to this effect [8]:

"*In the absence of consent, to comply with UK Data Protection legislation, a case report about a living person must be anonymised so that neither the individual, nor anyone who knows them, can identify themselves from the published article. The nature of case reports means that this is almost always impossible to achieve with certainty. Therefore, cases without consent for publication will not be considered*.

*If the person described in the case report has died, then consent for publication must be sought from their next of kin. If the next of kin are not traceable, and the authors have made every effort to trace the family, publication of the case may be possible if all three conditions specified by the Committee on Publication Ethics' (COPE) Code of Conduct are met*.

*If the individual described in the case report is a minor, or unable to provide consent, then consent must be sought from their parents or legal guardians. In these cases, the statement in the 'Consent' section of the manuscript should be amended accordingly*.

*Case reports without appropriate consent will be rejected prior to peer review*."

The most effective way to meet our policy continues to be via the consent form provided on our website, which is now available in nine languages [9]. The list of languages our consent form is available in will continue to be augmented on the advice of our authors.

In a very small number of cases we recognize that this new policy might preclude articles from publication. The protection of individuals in an open access, online environment must take precedence. We will work with authors to assist them to meet our policies and encourage any authors of case reports where consent cannot be obtained to contact our editorial office in advance of their submission.

## Authors' contributions

IH wrote the first draft of the manuscript; MRK was involved in its review and critical revision before submission. Both authors read and approved the final manuscript.
